# Maternal Risk Factors Associated with the Development of Cleft Lip and Cleft Palate in Mexico: A Case-Control Study

**Published:** 2017-07

**Authors:** Emmanuel Angulo-Castro, Luis F Acosta-Alfaro, Alma M Guadron-Llanos, Adrian Canizalez-Román, Fernando Gonzalez-Ibarra, Ignacio Osuna-Ramírez, Joel Murillo-Llanes

**Affiliations:** 1 *Department of Perinatology, The Women's Hospital, Secretariat of Health, 80127, Culiacan, Sinaloa,* * Mexico. *; 2 *CIASaP, School of Medicine* *, Autonomous University of Sinaloa, 80246, Culiacan, Sinaloa, Mexico.*; 3 *Research Department, The Women's Hospital, Secretariat of Health, 80127, Culiacan, Sinaloa,* * Mexico.*; 4 *Department of Internal Medicine, Gulf Coast Medical Center, Panama City, 32405, Florida, USA. *; 5 *Faculty of Biological and Chemical Sciences, Autonomous University of Sinaloa, 80000 Culiacán, SIN, Mexico.*

**Keywords:** Cleft lip, Cleft palate, Pregnancy Complications

## Abstract

**Introduction:**

Cleft lip and palate, the most common developmental deformity, is seen worldwide and the etiology involves a combination of genetic and environmental factors. The purpose of this study was to determine the maternal risk factors associated with the development of cleft lip and cleft palate.

**Materials and Methods:**

We conducted a case control study at the Women’s Hospital in Culiacan, Mexico. Medical records were analyzed, including patients who delivered babies with and without cleft lip and cleft palate from January 2010 to December 2015. Multiple variables were analyzed, including gestational age, weight at birth, the use of folic acid and multivitamins during pregnancy, smoking, alcohol abuse, the use of recreational drugs, history of sexually transmitted infections, marital status, socioeconomic status, education, and nutritional status.

**Results:**

We found that the maternal risk factors with the strongest association for the development of cleft lip and cleft palate were the following: patients who were not taking folic acid during pregnancy [OR 3.27, 95% CI 1.32-8.09], P=0.00; patients who were not taking vitamin supplementation during pregnancy [OR 2.6, 95% CI 1.19-7.27], P=0.02; smoking during pregnancy [OR 2.05, 95% CI 1.23-3.41], P=0.01; and alcohol abuse during pregnancy [OR 1.90, 95% CI 1.17-3.08], P=0.03.

**Conclusions:**

The main risk factors associated with the development of cleft lip and cleft palate in a Mexican population at the Women’s hospital in Culiacan, Sinaloa, Mexico were smoking, alcohol abuse, and patients not taking folic acid and multivitamins during pregnancy.

## Introduction

The clinical presentation of oral clefts is varied and can be classified as isolated cleft palate or cleft lip with or without cleft palate. The abnormality can involve the lip, the hard palate and/or soft palate and it also can be complete or incomplete, unilateral or bilateral. It is important to emphasize this distinction because it refers to their embryological origin. These are defects that occur during embryological development when fusion of the lateral and medial nasal processes with the anterior extension of the maxillary processes on either side fails, resulting in a cleft lip or a defective consolidation of mesenchymal palatine ridges (1-3).

Cleft lip (CL) is defined as a congenital anomaly characterized by an abnormal opening in the upper lip. It may be unilateral, predominantly on the left side, or bilateral, and it may be associated or not with cleft palate. The majority of affected children with CL have no association with other abnormalities. They are known to have a strong genetic component and therefore the risk of recurrence among siblings is possible. Genetic factors may interact with environmental factors such as smoking or use of illicit drugs during pregnancy. The presence of environmental risk factors may play an important role in the emergence of CL in newborns (4,5).

The incidence of oral clefts diagnosed at birth varies in every country, but may be as high as one in 700 newborns (6). In Europe the reported incidence is approximately 6 in 10,000 births; while in Asia and Canada these rates are doubled (7). In Mexico the reported incidence was 0.636 per 1,000 births in 2003, 0.479 in 2005 and 0.479 per 1000 newborns in 2006, making these abnormalities among the most common congenital malformations in newborns (8).

The diagnosis of oral cleft malformations can be done clinically at birth, but diagnosis is also possible during pregnancy using an obstetrical ultrasound. The recognition of these conditions is very important because of the comorbidities that are usually associated with them, such as speech and hearing abnormalities, psychological comorbidities and impaired social integration. Children with these disorders have a higher morbidity and mortality compared with normal individuals. Boulet et al. estimated that the health cost was eight times higher compared to children without these defects in the US (9,10).The Women’s Hospital of Culiacan, Sinaloa, Mexico started operating since 2009 and provides medical services to women and newborns with an average of 6,000 to 8,000 births per year. Unfortunately it has been observed that prenatal care and follow up is a major problem because only approximately 50% of all women have some king of follow up during pregnancy and only about 30 to 40% are on a multivitamin and folic acid regimen. This study was conducted in order to find the maternal risk factors associated with cleft lip and cleft palate in the population of our hospital. 

## Materials and Methods

A case-control study was conducted at The Women’s Hospital of Culiacan, Sinaloa, Mexico from 2010 to 2015. The study was done retrospectively by reviewing all medical records of newborns and mothers with (prevalent cases) or without (controls) the presence of cleft lip and cleft palate from the period of January 1^st^, 2010 to December 31^st^, 2015. We included patients whose medical records indicated that they came to the hospital, received medical care, and gave birth to a newborn with or without diagnosis of cleft lip or cleft palate. Exclusion criteria were patients without medical records, patients who were not treated at the Women’s Hospital in the study period and patients whose medical records were incomplete or did not have enough information. 

A total of 24 cases and 24 controls were enrolled in the study. The patients included were cases with a cleft lip (CL) and cleft palate (CP). The cases were matched to the controls on age and place of conception. The data collected from the medical records in the study groups were: age, weeks of gestation at delivery, weight at birth, the use of folic acid and multivitamins during pregnancy, smoking history during pregnancy, alcohol abuse, use of illicit drugs, history of sexually transmitted infections during pregnancy, marital status, socioeconomic level, education and nutritional status.

***Statistical Analysis***

Descriptive statistics were calculated by case and control groups. The frequencies and percentages for qualitative and numeric variables were obtained; measurements of central tendency and dispersion were also calculated and presented in tables. In order to compare quantitative variables, the T student test was used. In addition, the Kruskal-Wallis one-way analysis of variance was used depending on the type of distribution. We used multiple logistic regressions to estimate unadjusted and adjusted associations with a 95% confidence interval and p-values. The analysis was performed with SPSS version 15.0. 

## Results

A total of 42,911 births were registered during the period of 2010 to 2015 at The Women’s Hospital of Culiacan, Sinaloa, exico, from those, a total of 24 births with cleft lip andcleft palate were detected. The mean age of all mothers in the study population was 24.56±4.97 years, with a median age of 26 and 23 years for the cases and controls respectively (P=0.07). The mean weeks of gestation at birth were 37.54 ± 1.79 with a minimum of 33 and maximum of 41 weeks. The median gestational age was 38.5 in the case group and 37 in the control group (P=0.02). The mean weight at birth was 2,896 ±702.02 grams with a minimum weight of 1,420 grams and a maximum weight of 3,950 grams. The results obtained in regard of the body mass index, nutritional status according to the World Health Organization (WHO), marital status, educational level and number of births are presented in Table 1.

**Table 1:** Sociodemographic characteristics of mothers with and without children with cleft lip and cleft palate

	**Cases n(%)**	**Controls n(%)**	
Age (years)	26±5.22	23.12±4.11	P
Gestationage (weeks)	38	37	0.02
Birthweight (grams)	2,891	2,971	0.45
Nutritional status Underweight	6(25)	2(8.3)	0.1244
Normal	3(12.5)	11(45.8)	0.012
Overweight	7(29.16)	17(70.83)	0.004
Obesity	8(33.3)	4(16.6)	0.18
Agegroup (years)<20	4(16.6)	6(25)	0.47
20- 35	19(79.16)	18(75)	0.73
>35	1(4.16)	0(0)	0.31
Scholarship Primary education	5(20.83)	6(25)	0.73
High school education	7(29.16)	3(12.5)	0.15
Upper middle education	4(16.6)	10(41.66)	0.058
Highereducation	5(20.8)	5(20.8)	1.0
Illiterate	5(20.8)	0(0)	0.019
Civil status Single	6(25)	4(16.6)	0.47
Married	10(41.66)	12(50)	0.56
Concubinage	8(33.3)	8(33.3)	1.0
Parity First pregnancy	6(25)	11(45.8)	0.13
Second pregnancy	6(25)	12(50)	0.07
Third or more pregnancy	11(45.8)	1(4.16)	0.001

n=number, P=Statistical significance, %=percentage

* mean and standard deviation. Source: Medical records of female patients’ beneficiaries of popular insurance who attended the end of her pregnancy in the Women’s hospital of Culiacan, Sinaloa 2010-2015 period.

In regard to nutritional status, we found 8 women with obesity (33.83%) in the case group compared with 4(16.66%) in the control group (P=0.18). Illicit drug use during pregnancy occurred in 20.83% of the women in the case group and in 12.5% of controls (P=0.43). In the case group, 3(12.5%) were teenage mothers and 6(25%) were first pregnancy mothers. The history of sexually transmitted infections, nutritional status, and number of births is presented in Table 2.

**Table 2:** Frequency of sexually transmitted diseases in mothers with children with and without cleft lip and cleft palate

**Clinicalfeatures**	**Mothers of children with ** **cleft lip(n=24)**		**Mothers of children without cleft ** **lip(n=24)**	
STD duringpregnancy	**n**	**%**	**n**	**%**
Chlamydia	1	4.16	0	0
Gonorrhea	1	4.16	0	0
Herpes Simplex	1	4.16	1	4.16
Trichomoniasis	2	8.33	1	4.16
Human Papilloma Virus	2	8.33	1	4.16
Neither	17	70.92	21	87.52

STD= sexual transmission disease, n= number, %= percentage, Source: Medical records of female patients’ beneficiaries of popular insurance who attended the end of her pregnancy in the Women’s hospital of Culiacan, Sinaloa 2010-2015 period.

The unadjusted maternal risk factors with the greater association with cleft lip and cleft palate were: the lack of consumption of folic acid and multivitamins during pregnancy in 15 patients (62.5%) versus 4 patients (16.67%) in the case group and control group respectively [OR 3.27, 95% CI 1.32-8.09], and the history of smoking during pregnancy [non adjusted OR was 2.05, but adjusted OR by weight was 8.1, 95% CI 1.6-39.3, P=0.009]. These results are presented in Table 3. 

**Table 3:** Bivariate analysis between main study variables versus cleft lip presence

**Risk factor´s**	**Mothers of children with cleft lip(n=24)**		**Mothers of children without cleft lip(n=24)**		**OR**	**OR** *	**95% CI**	**P**
	n	%	n	%				
Folic acid intake during pregnancy								
Yes	9	37.5	20	83.3	3.27	--	1.32-8.09	0.00
No	15	62.5	4	16.67				
Consumption of vitamin supplements								
Yes	10	41.7	21	87.5	2.60	--	1.19-7.27	0.02
No	14	58.3	3	12.5				
Smoking								
yes	11	45.83	3	12.5	2.05	8.1	1.6-39.3	0.009
No	13	54.17	21	87.5				
Alcoholism								
Yes	8	33.3	2	8.33	1.9	--	1.17-3.08	0.03
No	16	66.67	22	91.67				
Use of drugs								
Yes	5	20.83	3	12.5	1.31		0.70-2.46	0.88
No	19	79.17	21	87.5				
History of Newborn with congenital disease								
Yes	2	8.33	0	0	2.09		1.54-2.82	0.02*
No	22	91.67	24	100				
New born gender								
Male	16	66.66	14	58.33	1.2		0.64-2.22	0.55
Female	8	33.34	10	41.67				


n= number, %= percentage, p=Statistical significance, OR= non adjusted Odds Ratio, OR

* = adjusted Odds Ratio CI= confidence interval 95%. Source: Medical records of female patients’ beneficiaries of popular insurance who attended the end of her pregnancy in the Women’s hospital of Culiacan, Sinaloa 2010-2015 period.

The specific type of congenital malformations in the patients with cleft palate and cleft lip are presented in Table 4.

**Table 4:** Type of congenital malformations in patients with cleft lip and cleft palate present in our population under study

**Congenital malformations type**	**No.**
Patent ductus arteriosus	1
Atrial septaldefect	1
Cryptorchidia	1
Esophageal atresia	1
Pulmonaryhypoplasia and dysplasia	1
Tracheoesophageal fistula	1
Syndactyly	1
Patau syndrome	1
Agenesis of the corpus callosum and hydrocephalus	2
Hypoplasia of the aortic arch	1
Agenesis of the nose	1
Microcephaly	1

Only in three patients had congenital malformations (12.5%)

## Discussion

Epidemiological and experimental studies suggest that maternal risk factors play a significant role in the development of cleft lip. The major risk factors include: smoking (or maternal exposure to second hand smoking), a maternal age less than 20 or older than 35 years (11), inter-pregnancy periods of less than two years, consanguinity, use of certain drugs during pregnancy such as anticonvulsants and anticoagulants, and exposure to infections during pregnancy including toxoplasmosis, rubella, cytomegalovirus and herpes virus (TORCH) (12,13). In the present study, we found the median age to be higher for the cases than the controls. 

In our study population, the major maternal risk factors associated with the development of cleft lip and cleft palate were lack of folic acid intake and multivitamin supplements during pregnancy, in addition to smoking and alcohol abuse during pregnancy. However, when adjusted according to the nutritional status of the mother, the smoking in both malnourished and obese mothers increased the risk for orofacial malformations when this variable was adjusted for body mass index. Moreover, alcohol consumption doubly increased the risk in undernourished mothersbut not in obese mothers, a result that differs from other studies as it has been shown previously that both obesity and low maternal weight are risk factors for cleft lip and cleft palate (14). 

A meta-analysis conducted by Goh Yi et al, concluded that the consumption of folic acid before and during pregnancy was associated with a decreased risk of several birth defects, including cleft lip and cleft palate. Another study by Wilcox et al, showed that folic acid consumption during pregnancy reduced the risk of cleft lip up to 30% with 400 micrograms per day or higher doses, adjusted for confounding variables such as smoking (15,16).

A study carried out by Cisneros et al, found that the history of smoking and alcohol consumption during pregnancy are major risk factors and even considered chemical teratogens during pregnancy, putting the fetus at a high risk for the development of multiple congenital abnormalities (17,18).

In Mexico, Garcia and Navarro reported that cleft lip ranked first among all congenital malformations with an incidence rate of 0.56 per 1,000 live births(8). The Annual National Epidemiological Surveillance reported a prevalence rate for cleft lip similar to our study in The Women’s Hospital of Culiacan(19), Sinaloa, Mexico (0.54 per 1,000 newborns). In Venezuela, in 2002, the incidence rate for cleft lip was 0.74 per 1000 live births and the prevalence was higher than the one observed in our study (18,20). 

In the present study, only 4.12% of mothers whose children were born with cleft lip had an association with various congenital malformations, and currently the national frequency rate varies from 4 to 20%. It is important to note that our city is surrounded by large agricultural areas where herbicides, insecticides, and fertilizers are often used (21). These chemicals could perhaps also cause genetic mutations in exposed pregnancies (22,23), but these are speculative questions th Epidemiology, Etiology, and Treatment of Isolated Cleft Palateat require further investigation.

Although the medical records data (all patients in each group) were reviewed carefully, the authors recognize that the present study possesses various weaknesses, including the small size of the sampleas well as the biases involved in a case-control study and the fact that the presence of polymorphisms or aneuploidies were not investigated in the newborns with cleft lip and cleft palate such as those pregnant women exposed to agrochemicals. However, to the best of our knowledge, this is the first study conducted in the northwest region of Mexico trying to determine the maternal risk factors for the development of cleft lip and cleft palate in newborns. 

## Conclusion

The main risk factors associated with the development of cleft lip and cleft palate in a Mexican population at the Women’s hospital in Culiacan, Sinaloa, Mexico were smoking, which adjusted by weight significantly increases the risk of orofacial malformations, alcohol abuse, and patients not taking folic acid and multivitamins during pregnancy. 
